# Fecal Microbiota Transplantation Improves the Quality of Life in Patients with Inflammatory Bowel Disease

**DOI:** 10.1155/2015/517597

**Published:** 2015-06-04

**Authors:** Yao Wei, Weiming Zhu, Jianfeng Gong, Dong Guo, Lili Gu, Ning Li, Jieshou Li

**Affiliations:** Institute of General Surgery, Jinling Hospital, Medical School of Nanjing University, Nanjing, Jiangsu 210002, China

## Abstract

*Introduction*. To determine the effect of fecal microbiota transplantation (FMT) on quality of life (QoL) in patients with inflammatory bowel disease (IBD). *Methods*. Fourteen IBD patients, including 11 Ulcerative colitis (UC) and 3 Crohn's disease (CD), were treated with FMT via colonoscopy or nasojejunal tube infusion. QoL was measured by IBD Questionnaire (IBDQ). Disease activity and IBDQ were evaluated at enrollment and four weeks after treatment. Patients' attitude concerning the treatment was also investigated. *Results*. One patient was excluded due to intolerance. All the other patients finished the study well. Mean Mayo score in UC patients decreased significantly (5.80 ± 1.87 versus 1.50 ± 1.35, *P* < 0.01). Mean IBDQ scores of both UC and CD patients increased (135.50 ± 27.18 versus 177.30 ± 20.88, *P* = 0.00063, and 107.33 ± 9.45 versus 149.00 ± 20.07, *P* = 0.024) four weeks after fecal microbiota transplantation. There was no correlation between the IBDQ score and Mayo score before and after FMT. Patients refused to take FMT as treatment repeatedly in a short time. *Conlusions*. Fecal microbiota transplantation improves quality of life significantly in patients with inflammatory bowel disease.

## 1. Introduction

Inflammatory bowel disease (IBD), an umbrella term for both Crohn's disease (CD) and Ulcerative colitis (UC), is thought to be caused by dysbiosis in the intestinal flora and aberrant activation of the mucosal immune system [1–3]. It is a lifelong disease that affects a person's social and psychological wellbeing, particularly if poorly controlled [4]. Meantime, costs, and risks of surgical or medical management options for patients must be weighed against the benefits provided. Therefore, most patients with IBD have impaired Health Related Quality of Life (HRQOL) compared to the healthy person. So, managing HRQOL in IBD is increasingly being considered as an important treatment goal and a simple way to measure utility to assess the “value-for-money” of different treatment options.

Based on the understanding of changes of intestinal flora in IBD patients, fecal microbiota transplantation (FMT) has been put forward to rebuild IBD patients' flora balance and to achieve therapeutic purposes [5]. The first report of the use of FMT for patients with UC was published in 1989. Since then, evidence has been accumulating of its usage in IBD patients. However, results concerning its usage are still inconclusive. In addition, patients' attitudes also influence the application of FMT in IBD. Although FMT was perceived as a “natural” and safe therapy compared to conventional therapies, the aesthetics of the treatment, previously reported issues with intolerance and need for repeated FMT hinder its usage in IBD. Few studies focused on whether it can affect QoL of IBD patients and patients' attitude towards FMT. The purpose of the current study is to explore the effect of FMT on the QoL of IBD patients and their attitudes towards FMT.

## 2. Materials and Methods

### 2.1. Patients

Patients admitted to the IBD center of Jinling Hospital from September 2013 to February 2014 (Table 1) were screened for eligibility. All patients had been previously diagnosed as IBD according to standard criteria (clinical, endoscopic, and histological). They all had been informed and agreed to undergo FMT and answer the Inflammatory Bowel Disease Questionnaire (IBDQ) as a measure of Health-Related Quality of Life (HRQOL). Patients' attitudes towards FMT were also investigated using three questions, especially about repeated FMT procedure.

This was an uncontrolled pilot clinical trial and it was approved by the Institutional Ethics Committee of Jinling hospital. Patients satisfying the following criteria were recruited: (1) age range of 18–70 years (for CD < 40); (2) a CDAI score of >150 and <400; or Mayo score (UCDAI) of 2–10 and (3) for CD who had a C-reactive protein (CRP) level of >10 mg/L at enrollment. The exclusion criteria were as follows: (1) if women are pregnant or intend to get pregnant during the study period, (2) if patients had end-stage disease, (3) if patients are participating in other clinical trials or participated in other clinical trials within 3 months prior to transplantation, (4) if infectious (viruses, bacteria, parasites, or other microorganisms) colitis occur, (5) if patients are scheduled for abdominal surgery, (6) if patients have taken probiotics/prebiotics/synbiotics/antibiotic/PPI orally in the last month, (7) if patients suffer from severe anemia (Hbg < 6 g/dL), heart cerebrovascular accident, and bypass or underwent stent implantation surgery in the last 6 months, (8) if patients had coagulation disorders or immune suppression status (defined as a history of opportunistic infections within one year, recurrent oral ulcers, multiple lymphadenopathy, neutropenia, etc.) or underwent major abdominal transplant surgery in the last 3 months, (9) if patients took TNF-*α* monoclonal antibody 2 months before transplantation or planned to take it within one month after transplantation, (10) if patients had a history of megacolon [6].

### 2.2. Donor

The standardized stool donor is a healthy unrelated adult (22 years old, female), living in a different household from the recipients. Donor had received no antibiotic therapy within the last 6 months. To avoid a transmission of other diseases, donor had to have a negative history for intestinal diseases or recent gastrointestinal infections, autoimmune or other immune-mediated diseases, or any kind of malignancies. Chronic hepatitis B and C, human immunodeficiency virus, and syphilis were excluded serologically and the donor's stool was tested for* C. difficile*,* Enterohemorrhagic Escherichia coli*,* Salmonella*,* Shigella*,* Yersinia*, and* Campylobacter* as well as parasites [7].

### 2.3. Donor Material Preparation

Donor produced stool samples within 6 hours before the scheduled FMT. 60 g fresh fecal samples were blended with 350 mL sterile saline for 10 minutes in a designated GI laboratory space. This blended fecal mixture was then filtered through 3 gauze pieces to remove larger sediments. Filtered fecal preparation was then kept at 4°C until FMT was administered.

### 2.4. Transplantation Procedure

Patients were maintained on vancomycin (500 mg orally, twice a day for three consecutive days) until 12 hours before FMT. The day before the procedure, patients took polyethylene glycol electrolyte powder to wash out fecal material [7]. The prepared donor material (300 mL) was administered via the colonoscope's biopsy channel in UC cases and via nasojejunal tube in CD.

### 2.5. Definition of Clinical Outcome

Patients were instructed to contact the doctor in case of symptom recurrence and followed up for four weeks after the procedure. An IBDQ score >170 was considered clinical remission and an increase of >16 means clinically meaningful improvement. As in the diseases activity scores, clinical response was defined as a decrease in CDAI score by >70 and clinical remission was defined as a CDAI score <150 and CRP level <10 mg/L in CD [8] and clinical response was defined as a decrease in Mayo score by >1; clinical remission was defined as Mayo score <2 in UC [9].

### 2.6. Patients' Attitude

Patients were asked to answer three questions for investigating their attitude towards FMT after follow-up visit.

The three questions were as follows:

1. Would you like to receive FMT repeatedly as a treatment measure when the disease relapses? Y/N?
2. Whose stool would you like to accept for FMT? Relatives or nonrelatives?
3. Which route would you like to receive FMT? Colonoscopy or enema or nasojejunal tube?

### 2.7. Statistical Analysis

No power calculation or sample size assessment was done for this pilot study. Statistical analysis was carried out with SPSS version 17.0. The outcomes before and after treatment were compared using the paired *t*-test. A regression model was used to explore the relationship between HRQOL and diseases activity scores. *P* < 0.05 was considered significant.

## 3. Results

### 3.1. Baseline Characteristics of Patients

Fourteen patients were enrolled, including 3 CD and 11 UC (male/female 6/8) (Table 1). The mean age is 43.46 ± 16.44 years. Patients continued to take the medication (5-ASA/steroid) with unchanged dose throughout the study period. Patient C had a terminal ileostomy before FMT, so we couldn't get a CDAI score; but the patient finished the IBD Questionnaire and we had reason to value it.

### 3.2. Intolerance and Adverse Events

One patient showed intolerance with FMT and immediately leaked donor material for about 80 mL within 30 min after the procedure. He was later proved to have low-grade intraepithelial neoplasia by pathological examination and was not included in the clinical response and IBDQ evaluation. Two patients reported a moderate degree of fever (the higher one was 38.3°) after FMT, but spontaneously normalized within 24 h. The remaining cases tolerated well without leakage for an average of 6 hours and no patients who finished FMT via nasojejunal tube complained about abdominal distension or other discomfort. No serious adverse events were noted.

### 3.3. Clinical Response to FMT and Evaluation of IBDQ

**Figure 1:**
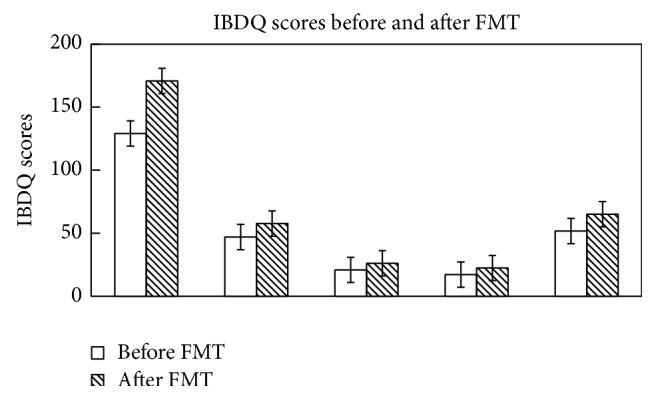
Changes of Inflammatory Bowel Disease Questionnaire score before and 4 weeks after fecal microbiota transplantation.

All the patients had a remission of the symptoms like blood in stool, fecal urgency, and diarrhea after four weeks of FMT; and there were significant improvements in total IBDQ scores after FMT. In UC cases, the mean Mayo score decreased from 5.80 ± 1.87 to 1.50 ± 1.35 (*P* < 0.01) and the mean IBDQ increased from 135.50 ± 27.18 to 159.00 ± 26.11 (*P* = 0.032) two weeks after FMT and to 177.30 ± 20.88 (*P* = 0.00063) four weeks after FMT. In CD cases, the mean CDAI score decreased from 345.00 ± 77.78 to 135.00 ± 7.07 (*P* = 0.082) and the mean IBDQ score increased from 107.33 ± 9.45 to 127.67 ± 14.57 (*P* = 0.067) two weeks after FMT and to 149.00 ± 20.07 (*P* = 0.024) four weeks after FMT (Figure 1).

### 3.4. Correlation between UC-IBDQ and Mayo Score

**Figure 2:**
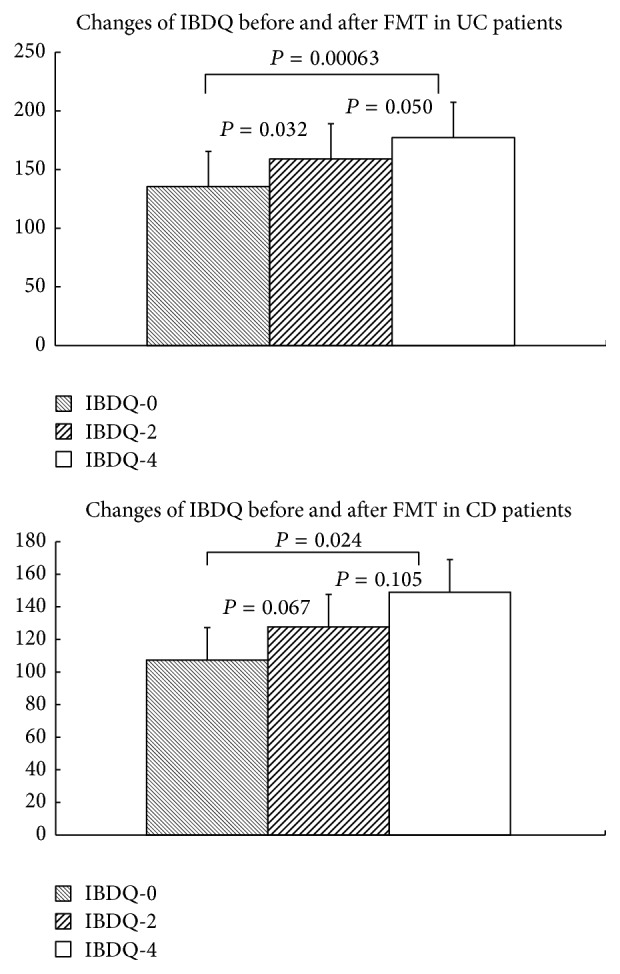
Mean Inflammatory Bowel Disease Questionnaire score before and after two and four weeks fecal microbiota transplantation.

**Figure 3:**
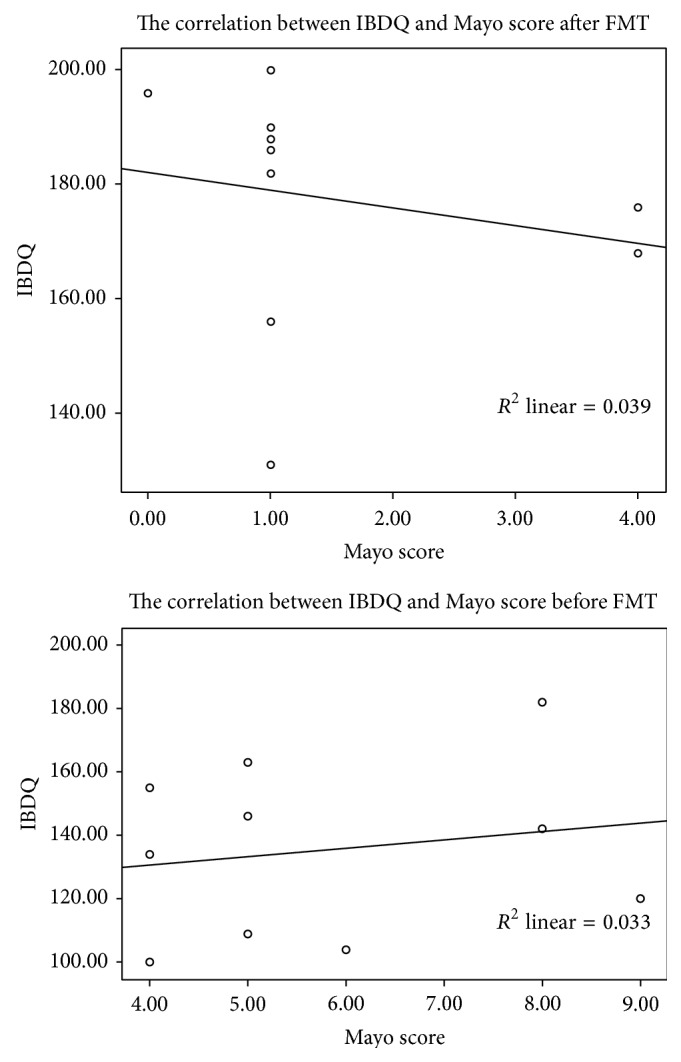
Linear correlation between the Inflammatory Bowel Disease Questionnaire score and Mayo score before and four weeks after fecal microbiota transplantation.

In UC cases, 6 of 7 (85.71%) patients with the total IBDQ score >170 achieved clinical remission, and the mean change was 40.33 ± 17.14 after treatment. Three UC patients did not have a total IBDQ score >170, but the mean change achieved was 47.33 ± 16.50 and they all reported that they had a clinically meaningful change of symptoms. No CD patients achieved an IBDQ total score >170 four weeks after FMT (Figure 3). There was no correlation between the IBDQ and Mayo score before and after FMT (*R* ^2^ = 0.033/0.039, *P* = 0.583/0.617) (Figure 2). We did not calculate the correlation between IBDQ and CDAI both before and after treatment as there were only 3 CD patients.

### 3.5. Patients' Attitude towards FMT

All patients considered FMT as an alternative method to treat the disease but they expressed that they may not receive FMT repeatedly in a short time because FMT must be carried out via colonoscope which needs intestinal preparation or by relying on a tube which makes them feel uncomfortable; and they eagerly hope for a colorless and odorless microbiota pill to appear. Most of them prefer colonoscope to other ways and there was no bias between relatives and nonrelatives on the choice of donor (Table 2).

## 4. Discussion

IBD is a chronic relapsing inflammatory disorder of the intestine. The etiology is unknown, but interactions between gut microbiota and the host seem to be involved in pathogenesis. The intestinal microbiota of patients with IBD has reduced diversity compared with that of healthy populations, based on studies reporting 25% fewer microbial genes [10, 11]. A hypothesis suggests that IBD results from continuous antigenic stimulation by nonpathogenic commensals, leading to an exaggerated sustained immune response in genetically predisposed hosts [12]. In addition, IBD patients are also more likely to have been prescribed antibiotics in the 2–5 years preceding their diagnosis. Thus, it seems reasonable that restoration of a healthy balance of intestinal flora by FMT could be therapeutic for IBD.

FMT for Ulcerative colitis has been described in several publications which showed complete resolution of all symptoms even cessation of medications without relapse. Recent studies have confirmed these findings; a meta-analysis of FMT for patients with IBD found that 63% of patients with UC entered remission, 76% were able to stop taking medications for IBD, and 76% experienced a reduction in gastrointestinal symptoms [5]. Measurement of quality of life is especially pertinent in chronic diseases that have periods of activity and remission and for which there is no cure, as is the case in IBD [13]. Therefore, the quality of life should be taken into account when evaluating the results of different treatments in subjects with this type of disease, and restoring previous quality of life should be one of the therapeutic objectives; but only two studies come down to the quality of life in IBD.

The results of this study demonstrated that performing FMT is feasible and it is well tolerated and safe in IBD adults. The study also indicates the potential efficacy of FMT in IBD. At the same time, we discussed the quality of life intensively. While given FMT once, patients' HRQOL improved significantly in UC patients. In CD patients, the IBDQ score did not increase significantly two weeks after FMT but it was meaningful four weeks later. We report, for the first time, using FMT to mitigate a human condition and improve the HRQOL; but IBDQ lacks a description of patients' feelings about the intervention. Therefore, we investigated patients' attitudes towards FMT. Our patients showed that they were willing to receive this new method despite its unappealing nature, for its better to have FMT than to be tortured by the disease. Kahn et al. [14] also revealed that both adult patients with UC and parents of UC children are willing to consider FMT as treatment.

Intestinal decontamination preparation before FMT is used in the current study. Most references and published papers used vancomycin for patients with* Clostridium dificile* Infections (CDI). Rifaximin and metronidazole were also reported as drugs for intestinal preparation. As there is no standard protocol for recipient management in UC prior to the initiation of the study, we referred to the protocols for CDI and employed most used vancomycin for intestinal decontamination preparation.

In our study, no CD patients achieved an IBDQ total score >170 four weeks after FMT; but the ΔIBDQ score in 66.67% (2/3) CD patients was ⩾50. This may be because of the small number of patients and short duration of observation after FMT and the patients' poor condition at the beginning (lower BMI and higher CDAI score). Providing these patients with FMT repeatedly might get better results.

There are several limitations in this study. First, we had no control groups, and it was not blinded. In addition, it was a single-center trial, the number of patients was small, and patients were from the same nation. Cultural expectations may influence patients' IBDQ score, especially the dimensions of social function and emotional status, which may affect the generalization of the present study.

## 5. Conclusion

Fecal microbiota transplantation improves life quality in patients with inflammatory bowel disease.

## Figures and Tables

**Table 1:** Basic characteristics of patients.

Patients	Sex	Age	Diagnosis	Basic IBDQ/activity	Duration of disease (year)	Concomitant medication	Route of FMT	BMI
A	Female	70	UC	104/6	10	Mesalazine	C	19.4
B	Female	59	UC	120/9	4	Mesalazine glucocorticoid	C	23.9
C	Male	40	CD	100/—	6	SASP	NJT	16.7
D	Male	16	CD	118/400	0.5	None	NJT	12
E	Female	48	UC	162/8	3	Mesalazine	C	24.2
F	Female	47	UC	100/4	6	Gentamicin	C	18.3
G	Male	24	CD	104/290	1	None	C	17.6
H	Male	26	UC	109/5	2	Mesalazine	C	20.8
I	Female	39	UC	155/4	1.5	Gatifloxacin	C	22.3
J	Female	49	UC	142/8	10	Mesalazine	C	23.2
K	Female	70	UC	163/5	1	Norfloxacin	C	20
L	Male	36	UC	134/4	6	None	C	26.1
M	Male	41	UC	123/12	1	Mesalazine	C	22.8
N	Female	32	UC	146/5	5	Mesalazine	C	21.5

SASP: salicylazosulfapyridine; UC: Ulcerative colitis; CD: Crohn's disease; C: colonoscope; NJT: nasojejunal tube.

**Table 2:** Patients' attitude towards FMT.

If repeated FMT in short time is needed	
Yes	3 (21.43%)
No	11 (78.57%)
The donor	
Relatives	6 (42.86%)
Nonrelatives	8 (57.14%)
The transplantation path	
Colonoscope	7 (50%)
Enema	3 (21.43%)
Nasointestinal tube	4 (28.57%)

## Abbreviations

IBDQ: Inflammatory Bowel Disease Questionnaire
FMT: Fecal microbiota transplantation
UC: Ulcerative colitis
CD: Crohn's disease.
